# Association of microRNA-7 and its binding partner *CDR1-AS* with the prognosis and prediction of 1^st^-line tamoxifen therapy in breast cancer

**DOI:** 10.1038/s41598-018-27987-w

**Published:** 2018-06-25

**Authors:** K. Uhr, A. M. Sieuwerts, V. de Weerd, M. Smid, D. Hammerl, J. A. Foekens, J. W. M. Martens

**Affiliations:** Erasmus MC Cancer Institute, Erasmus University Medical Centre, Department of Medical Oncology and Cancer Genomics, Wytemaweg 80, 3015 CN Rotterdam, The Netherlands

## Abstract

The large number of non-coding RNAs (ncRNAs) and their breadth of functionalities has fuelled many studies on their roles in cancer. We previously linked four microRNAs to breast cancer prognosis. One of these microRNAs, *hsa-miR-7*, was found to be regulated by another type of ncRNA, the circular non-coding RNA (circRNA) *CDR1-AS*, which contains multiple *hsa-miR-7* binding sites. Based on this finding, we studied the potential clinical value of this circRNA on breast cancer prognosis in a cohort based on a cohort that was previously analysed for *hsa-miR-7* and in an adjuvant hormone-naïve cohort for 1^st^-line tamoxifen treatment outcomes, in which we also analysed *hsa-miR-7*. A negative correlation was observed between *hsa-miR-7* and *CDR1-AS* in both cohorts. Despite associations with various clinical metrics (e.g., tumour grade, tumour size, and relapse location), *CDR1-AS* was neither prognostic nor predictive of relevant outcomes in our cohorts. However, we did observe stromal *CDR1-AS* expression, suggesting a possible cell-type specific interaction. Next to the known association of *hsa-miR-7* expression with poor prognosis in primary breast cancer, we found that high *hsa-miR-7* expression was predictive of an adverse response to tamoxifen therapy and poor progression-free and post-relapse overall survival in patients with recurrent disease.

## Introduction

Non-coding RNAs (ncRNAs)^1^ are defined as genes that are transcribed into RNAs, but not translated into proteins, and are not structural RNAs like tRNA or rRNA^2^. MicroRNAs, which regulate mRNA translation^3^, are likely the best studied subgroup of ncRNAs; however, apart from this family is the recently recognised large group of long non-coding RNAs (lncRNAs)^4^. These lncRNAs are over 200 nt long^5^ and have a multitude of mechanisms that affect cellular activity, e.g., (1) by influencing the accessibility of genes to the transcriptional machinery by interacting with chromatin modifiers^6^; (2) by supporting DNA looping, promoter binding and activator/transcription factor recruitment, which can increase gene expression^4,6^; (3) by influencing mRNA splicing and mRNA stability and increasing translation^6^; and (4) by influencing protein phosphorylation, methylation and stability^6^.

Importantly, it has been recognised that specific lncRNAs can also interfere with microRNA functionality via several binding sites on their sequence, soaking up target microRNAs like a sponge^7^. Interestingly, microRNA binding does not necessarily lead to the degradation of a particular lncRNA^7^. One recently described lncRNA of this type is *CDR1-AS*, a circular RNA (circRNA)^7^, which is also known as *ciRS-7*, *CDR1as* or *CDR1NAT*, that specifically binds human microRNA-7 (*hsa-miR-7*) via 73 binding sites^7^. This interaction is conserved between human and mouse and probably other species as well^7^. *CDR1-AS* is generated from a linear transcript, following the general observation that circRNAs are spliced from longer transcripts and it shares a promoter with the lncRNA *LINC00632*, which is about 50x less abundant than the circRNA^8^.

CircRNAs are generally characterised as having high stability (transcript half-life), which is likely due to their resistance to exonucleases because of their circular conformation^9^. Furthermore, it has been shown that circRNAs show tissue-specific expression^10^ and are involved in cellular differentiation^11^ and pluripotency^12^. CircRNAs have a diverse range of functions, including influencing transcription, splicing and the translation of their host genes; serving as scaffolds for enzymes and substrates to enhance reaction kinetics and co-localization; functioning as protein sponges for RNA-binding proteins that can influence protein decoy; acting as microRNA sponges; and expressing peptides under rare circumstances^13^. At this point, there are several known mechanisms of circRNA biogenesis from linear transcripts, besides factors influencing their splicing^13^. It has been shown that reverse-complement ALU repeats are located to the right and left of the circularised sequence in some cases^13^. Furthermore, splicing factors have been shown to bind in the vicinity of encoded circRNAs, while in other cases, a lariat structure has been observed as a precursor molecule that includes the exon that was later identified to be a circRNA^13^. Finally, a less common mechanism involves RNA-binding proteins that are located further away from the circRNA, which can be classified as trans-acting factors^13^.

In regard to expression in malignancies, circRNAs have been found to show abnormal expression in haematological cancers as well as in several solid tumour types, such as colorectal cancer, lung cancer, kidney cancer, liver cancer, bladder cancer, gastric cancer, prostate cancer, CNS tumours, ovarian cancer and breast cancer^13^.

The multi-facetted microRNA *hsa-miR-7* is known to play roles in the differentiation of the intestinal epithelium^14^ and regulation of β-cell proliferation^15^, as well as to influence toll-like receptor 9 growth signalling in lung cancer cells^16^ and photoreceptor development in Drosophila^17^. *Hsa-miR-7* has also been implied in several cancer types, including lung cancer^18^, renal cancer^19^ and colorectal cancer^20^.

In 2008, we discovered that *hsa-miR-7* was a prognostic marker in hormone receptor-positive breast cancer^21^. Considering the fact that *CDR1-AS* is now recognised to be an *hsa-miR-7* sponge^7^, we addressed whether *CDR1-AS* expression was related to *hsa-miR-7* expression and, if so, whether its expression was also associated with breast cancer prognosis. To determine this, we used a cohort of patients with lymph node-negative (LNN) disease who did not receive any type of systemic adjuvant treatment to study the link with pure prognosis, i.e., the natural course of the disease (hereafter also named prognostic cohort). Additionally, to determine whether *CDR1-AS* and/or *hsa-miR-7* expression may have predictive value, we studied their relationship with the response to tamoxifen therapy and the lengths of progression-free survival (PFS) and post-relapse overall survival (PR-OS) in an adjuvant hormone-naïve cohort of patients who received tamoxifen as a 1^st^-line treatment for recurrent disease (hereafter named predictive cohort).

## Results

### Cell lines

**Figure 1:**
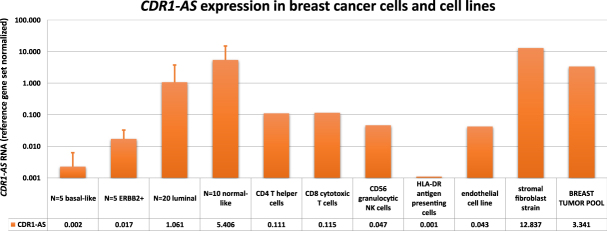
*CDR1-AS* expression in breast cancer cells and cell lines. Normalised *CDR1-AS* expression is shown in different breast cancer cell line subtypes (basal-like, ERBB2+, luminal and normal-like) on a logarithmic scale. Variations in expression levels within one subtype group are displayed as the standard deviation using error bars. N indicates the number of cell lines assessed per subtype group. Aside from the breast cancer cell lines, the expression is also shown for several immune cell types (CD4+ T helper cells, CD8+ cytotoxic T cells, CD56+ granulocytic NK cells and HLA-DR+ APCs), an endothelial cell line, a stromal fibroblast strain and a sample consisting of pooled material from different breast tumours. The HLA-DR+ APCs were from donor A, all other immune cells are from donor B. Of note is that the CD8+ T cells unfortunately showed a lower purity (61%) in contrast to the other immune cells (75–92% purity). The measured expression value is listed below each sample type.

To determine whether *CDR1-AS* is variably expressed, we initially measured *CDR1-AS* expression in several breast cancer cell lines, a breast tumour pool, an endothelial cell line, a stromal fibroblast strain, and several sorted immune cells of healthy donors (for included specimens, see Supplementary Fig. 1). This analysis showed that *CDR1-AS* was differentially expressed among the breast cancer cell line subtypes (see Supplementary Table S1), with higher *CDR1-AS* levels in the luminal and normal-like subtypes (Fig. 1). *CDR1-AS* expression was not only confined to breast cancer cells but was also detectable in other cell types present in tumours, as shown by the *CDR1-AS* expression in the endothelial cell line, the T cells, the granulocytic NK cells and the fibroblast strain, with especially high expression levels in the fibroblast strain derived from a patient’s stroma (Fig. 1). Furthermore, *CDR1-AS* was not only expressed by the cultured cell lines but was also present in a pooled sample containing cDNA from 100 different breast tumours. Altogether, the variable *CDR1-AS* expression provided a rationale to proceed with our study on the clinical relevance of this circRNA in our two breast cancer cohorts.

### *Hsa-miR-7* and *CDR1-AS* expression and prognosis

The first cohort evaluated for *CDR1-AS* expression consisted of RNA isolated from primary tumours from LNN breast cancer patients, including oestrogen receptor (ER, *ESR1*)-positive and ER-negative patients, who did not receive systemic adjuvant therapy (n = 345) (see the REMARK diagram in Supplementary Fig. 1) (prognostic cohort). This cohort included some of the ER-positive breast cancers in which we originally discovered that high *hsa-miR-7* expression was a marker for worse prognosis^21^ (see Supplementary Table S2 and Supplementary Fig. 1 for details). The patient overlap with our previous study was substantial (244 out of the n = 345 samples included in this study). The number of patients included in this study differed from our previous study because of the inclusion of additional available eligible patients (n = 101) as well as the exclusion of patients due to the lack of RNA or available qPCR data (n = 55). Furthermore, due to the differences in the ER expression measurement methods (mRNA- versus protein-based), the classification of some patients differed between the two studies (see Supplementary Table S2). As *CDR1-AS* has been shown to counteract *hsa-miR-7*^7^, we determined the association between *hsa-miR-7* and *CDR1-AS* in the 345 primary breast tumours from this prognostic cohort (Table 1). We observed an inverse correlation with *CDR1-AS* expressed at higher levels in the tumours with lower *hsa-miR-7* expression levels (Spearman rs −0.245, *P* < 0.0001).

**Table 1:** Associations of *hsa-miR-7* with *CDR1-AS* and clinical parameters in the prognostic patient cohort.

Parameters	n	*CDR1-AS*		*hsa-mir-7*	
		median expression [IQR]	*P*	median expression [IQR]	*P*
All patients	345	4.97 [8.74]		0.034 [0.053]	
***hsa-miR-7*** **expression**					
1st quantile (low)	173	7.16 [10.02]			
2nd quantile (high)	172	3.52 [6.78]	<***0.001***		
**Age at time of surgery (years)**					
≤40	38	3.37 [8.88]		0.039 [0.044]	
>40-≤55	134	5.09 [7.84]		0.032 [0.057]	
>55-≤70	117	5.65 [9.04]		0.033 [0.050]	
>70	56	4.46 [8.26]	*0.37*	0.047 [0.063]	*0.58*
**Menopausal status**					
Premenopausal	153	4.69 [8.31]		0.033 [0.057]	
Postmenopausal	192	5.19 [8.90]	*0.84*	0.035 [0.057]	*0.74*
**Pathological tumour size**					
pT1	161	6.95 [9.31]		0.029 [0.042]	
pT2+ unknown	171	3.97 [7.14]		0.037 [0.066]	
pT3+ pT4	13	3.66 [13.7]	***0.013***	0.055 [0.032]	***0.024***
**Tumour grade**					
Poor	197	4.72 [8.07]		0.043 [0.062]	
Unknown	95	3.58 [7.87]		0.033 [0.051]	
Moderate/Good	53	8.63 [7.87]	***0.001***	0.023 [0.027]	<***0.001***
**Tumour cell content**					
30–70%	230	6.10 [9.38]		0.033 [0.053]	
>70%	115	3.48 [7.78]	<***0.001***	0.042 [0.061]	*0.08*
Hormone receptor/growth factor status (RT-qPCR)*					
*ESR1*-negative	120	4.21 [7.84]		0.046 [0.062]	
*ESR1*-positive	225	5.41 [8.92]	*0.10*	0.030 [0.047]	***0.007***
*PGR-*negative	158	4.21 [7.84]		0.046 [0.066]	
*PGR*-positive	187	5.41 [9.47]	*0.08*	0.028 [0.045]	<***0.001***
*ERBB2*-non-amplified	294	5.49 [9.07]		0.033 [0.054]	
*ERBB2*-amplified	51	3.97 [6.17]	*0.22*	0.039 [0.059]	*0.43*
*EGFR* quantile 1 (low)	115	2.84 [4.85]		0.042 [0.062]	
*EGFR* quantile 2 (in between)	116	7.61 [9.61]		0.031 [0.059]	
*EGFR* quantile 3 (high)	114	6.24 [10.12]	<***0.001***	0.032 [0.046]	***0.042***

For the analysis on the association between *hsa-miR-7* expression and *CDR1-AS* expression, patients were divided into two equally sized groups based on median *hsa-miR-7* expression level. Next to this analysis result, the associations of the clinical parameters with *hsa-miR-7* and *CDR1-AS* gene expression are listed. The median *CDR1-AS* and *hsa-miR-7* expression levels per subcategory, including the interquartile range (IQR) are given additionally. Pathological tumour size is defined as follows: pT1 < = 2 cm, pT2 > 2 cm and < = 5 cm, pT3 > 5 cm, and pT4 = tumour with direct extension to chest wall and/or skin. *Cut-offs for positive and negative hormone receptor/growth factor status established as previously described^56,57^. *P* = p-value and n = number of patients. Significant p-values are printed in bold.

#### Association of *hsa-miR-7* and *CDR1-AS* with clinical variables

We next assessed whether *CDR1-AS* expression associated with the same traditional prognostic clinical parameters as *hsa-miR-7* did. No association was found for both markers with age, menopausal status or *ERBB2* status, while only *hsa-miR-7* was negatively associated with *ESR1* and progesterone receptor (*PGR*). By contrast, both markers were associated, although in opposite directions, with tumour size and tumour grade, indicating that high *CDR1-AS* levels were associated with good prognosis characteristics and high *hsa-miR-7* levels with poor prognosis characteristics. Additionally, *CDR1-AS* expression was found to be higher in tumours with a tumour cell content below 70%, i.e., tumours with a higher number of immune or stromal cells (Table 1).

A well-known target of *hsa-miR-7* is the epidermal growth factor receptor (*EGFR*)^22^. We therefore also assessed whether *CDR1-AS* might show a positive association with *EGFR* mRNA expression, and this was the case. The results showed higher *CDR1-AS* (and lower *hsa-miR-7*) expression in *EGFR*-high expressing tumours (Table 1).

#### *Hsa-miR-7* and *CDR1-AS* in metastasis-free survival (MFS) and overall survival (OS) in the prognostic patient cohort

We next studied whether the length of metastasis-free survival time (MFS) was correlated with *hsa-miR-7* and/or *CDR1-AS* expression. In the Cox univariate regression analysis, high *hsa-miR-7* levels were associated with poor prognosis in this cohort, including 225 ER-positive and 120 ER-negative patients, while *CDR1-AS* expression was not associated (Table 2).

**Table 2:** Univariate association of *CDR1-AS* and *hsa-miR-7* with MFS in the prognostic cohort.

Clinical parameters	CDR1-AS				hsa-miR-7			
	n	HR	(95% CI)	*P*	n	HR	(95% CI)	*P*
All patients	345	1.02	(0.90–1.16)	*0.76*	345	1.14	(1.00–1.30)	***0.043***
Hormone receptor/growth factor expression (RT-qPCR)*								
*ESR1*-negative	120	1.10	(0.83–1.45)	*0.50*	120	1.03	(0.77–1.37)	*0.86*
*ESR1*-positive	225	0.98	(0.84–1.12)	*0.31*	225	1.21	(1.06–1.38)	***0.006***

The association of *CDR1-AS*/*hsa-miR-7* expression and MFS in all patients and the patient subgroups based on *ESR1* expression is displayed. * Cut-offs for positive and negative hormone receptor/growth factor status were established as previously described^56,57^. n = number of patients, HR = hazard ratio, CI = confidence interval, and *P* = p-value. Significant p-values are printed in bold.

As ER-positive and ER-negative breast cancers can be considered two different diseases (due to different treatment requirements^23^ and profound differences in gene expression^24^), we also evaluated whether our genes of interest showed an association with MFS in the respective ER subgroups. High *hsa-miR-7* levels were strongly associated with a shorter MFS in ER-positive patients, but not in ER-negative patients, while *CDR1-AS* expression was not associated with the length of MFS in either ER subgroup (Table 2). Similar to our previous study^21^, *hsa-miR-7* (split at the median expression level) remained an independent biomarker for poor prognosis among ER-positive patients after correcting for the traditional prognostic factors: age, menopausal status, tumour size, grade, *ESR1*, *PGR* and *ERBB2* status (HR = 1.53, 95% CI 1.02–2.31, *P* = 0.04). Finally, we also related *hsa-miR-7* and *CDR1-AS* expression to the length of overall survival time (OS) in the complete prognostic cohort. Using Cox univariate regression analysis, among the two genes, only *hsa-miR-7* expression was associated with the OS length, with high *hsa-miR-7* levels being predictive of an earlier death (Table 3). However, this finding was not significant in the multivariate analysis (*P* > 0.05) (Table 3). In the ER-positive subset *hsa-miR-7* was associated with OS, a finding which remained significant in the multivariate analysis, including the traditional clinical factors (HR = 1.19, 95% CI 1.01–1.42, *P* = 0.041) (cohort size was n = 225 in the univariate analysis and n = 213 in the multivariate analysis due to the lack of some clinical data).

**Table 3:** Uni- and multivariate association of overall survival (OS) with *CDR1-AS*, *hsa-miR-7* and other clinical variables in the prognostic cohort.

Parameters	univariate model n = [345]				multivariate model [n = 331]			
	n	HR	(95% CI)	*P*	n**	HR	(95% CI)	*P*
	345							
**Age at time of surgery (years)**								
≤40 years	45	1			43	1		
41–50 years	96	0.94	(0.57–1.54)	*0.80*	94	1.10	(0.64–1.87)	*0.74*
51–70 years	159	1.14	(0.72–1.80)	*0.58*	152	1.29	(0.60–2.75)	*0.51*
>70 years	45	0.93	(0.49–1.77)	*0.83*	42	0.97	(0.37–2.52)	*0.95*
**Menopausal status**								
Premenopausal	153	1			149	1		
Postmenopausal	192	1.11	(0.82–1.51)	*0.50*	182	0.93	(0.49–1.78)	*0.84*
Pathological tumor size**								
≤2 cm	155	1			155	1		
>2 cm	176	1.30	(0.95–1.78)	*0.10*	176	1.31	(0.94–1.83)	*0.11*
**Tumour grade**								
Poor	197	1			194	1		
Unknown	95	1.01	(0.71–1.44)	*0.94*	85	0.75	(0.64–1.38)	*0.75*
Moderate/Good	53	0.74	(0.47–1.18)	*0.21*	52	0.58	(0.53–1.42)	*0.58*
Hormone/growth factor receptors*								
ER (log continuous *ESR1*)	345	0.99	(0.94–1.05)	*0.88*	331	1.08	(0.99–1.18)	*0.08*
PR (log continous *PGR*)	345	0.95	(0.89–1.00)	*0.07*	331	0.90	(0.82–0.98)	***0.020***
HER2 (*ERBB2*) status								
Not amplified	294	1			280	1		
amplified	51	1.44	(0.97–2.14)	*0.07*	51	1.51	(1.00–2.27)	***0.048***
					***hsa-miR-7*** **separately added to the base model**			
***CDR1-AS*** **(log continuous)**	345	1.03	(0.90–1.17)	*0.69*				
***hsa-miR-7*** **(log continuous)**	345	1.16	(1.02–1.32)	***0.028***	331	1.12	(0.97–1.29)	*0.11*
≤median	173	1			165	1		
>median	172	1.42	(1.04–1.92)	***0.025***	166	1.37	(0.98–1.92)	*0.07*

The association of *CDR1-AS* and *hsa-miR-7* gene expression with overall survival is displayed for the prognostic cohort. The univariate model is on the left side of the table and the multivariate model is displayed on the right side. *Cut-offs for positive and negative hormone receptor/growth factor status were established as previously described^56,57^. **In these analyses, clinical data are missing, and therefore, the included patients do not add up to 345. n = number of patients, HR = hazard ratio, CI = confidence interval, *P* = p-value. Significant p-values are highlighted in bold.

### *Hsa-miR-7 and CDR1-AS* in the recurrent breast cancer cohort

Based on the prognostic value of *hsa-miR-7* in the ER-positive patients, we were interested to assess whether the two ncRNAs could be predictive factors for the type of response to 1^st^-line tamoxifen therapy, PFS or PR-OS in recurrent disease. For these analyses, we had data available from 188 hormone-naïve breast cancer patients with ER-positive primary tumours who received tamoxifen as 1^st^-line therapy for recurrent disease (predictive cohort). Among this cohort, 39 patients had been included in our previous study^21^ (see also Supplementary Fig. 1).

#### Association of *hsa-miR-7* and *CDR1-AS* with clinical parameters in the predictive cohort

As 91 patients in this cohort were also included in our prognostic cohort (discussed in section B; see also Supplementary Fig. 1) and the overlap is therefore substantial, we will only discuss associations with clinical parameters that have not been discussed for the prognostic cohort or show a different result (all associations assessed are listed in Table 4). The *hsa-miR-7* and *CDR1-AS* expression levels were once again inversely correlated with one another (Spearman rs −0.277, P < 0.0001). In the analysis with the clinical parameters, *hsa-miR-7* was significantly positively associated with tumour cell content. In the predictive cohort, the pathological tumour size was not significantly associated with the two markers; similar results were noted for the nodal status with *CDR1-AS* and *PGR* expression with *hsa-miR-7*. However, the variable “dominant site of relapse” showed an association with *CDR1-AS* expression with the lowest expression in the primary tumours metastasising to distant sites other than local regional or bone.

**Table 4:** Associations of *CDR1-AS* and *hsa-miR-7* with clinical parameters in the predictive patient cohort.

Parameters	n	*CDR1-AS*		*hsa-mir-7*	
		median expression [IQR]	*P*	median expression [IQR]	*P*
All patients with *ESR1*-positive primary tumours	188	4.45 [6.42]		0.042 [0.077]	
**Age in years at start of 1** ^**st**^ **-line treatment**					
≤55	75	4.52 [5.82]		0.042 [0.083]	
56–70	67	3.84 [5.40]		0.040 [0.067]	
>70	46	4.35 [7.79]	*0.65*	0.045 [0.076]	*1.00*
**Menopausal status at start of 1** ^**st**^ **-line treatment**					
Premenopausal	53	4.69 [5.68]		0.046 [0.099]	
Postmenopausal	135	4.36 [6.91]	*0.4*	0.042 [0.069]	*0.66*
**Adjuvant chemotherapy**					
No	153	4.35 [6.87]		0.045 [0.086]	
CMF	21	4.82 [4.44]		0.033 [0.032]	
Anthracycline containing	14	4.36 [4.41]	*0.24*	0.047 [0.080]	*0.18*
**Tumour cell content**					
30–70%	111	4.98 [6.77]		0.033 [0.077]	
>70%	77	2.55 [5.22]	<***0.001***	0.049 [0.079]	***0.025***
**Pathological tumour size**					
pT1	53	4.85 [7.77]		0.038 [0.059]	
pT2 + unknown	116	4.39 [6.23]		0.040 [0.082]	
pT3 + pT4	19	4.55 [4.79]	*0.63*	0.078 [0.225]	*0.16*
**Tumour grade**					
Poor	104	3.90 [5.35]		0.048 [0.084]	
Unknown	57	4.12 [6.71]		0.040 [0.067]	
Moderate/Good	27	6.07 [10.42]	*0.093*	0.034 [0.073]	*0.35*
**Nodal status**					
Negative	91	5.00 [7.49]		0.045 [0.068]	
Positive	67	3.73 [6.08]		0.034 [0.068]	
Positive (tumour outside lymph nodes)	30	3.52 [4.93]	*0.085*	0.077 [0.275]	***0.024***
Hormone/growth factor receptors*					
*PGR-*negative	35	3.31 [8.08]		0.060 [0.235]	
*PGR*-positive	153	4.47 [6.05]	*0.26*	0.038 [0.073]	*0.21*
*ERBB2*-non-amplified	165	4.42 [6.65]		0.044 [0.086]	
*ERBB2*-amplified	23	4.66 [5.48]	*0.54*	0.029 [0.047]	*0.14*
**Disease-free interval before start 1** ^**st**^ **-line tamoxifen**					
≤1 year	76	4.67 [6.35]		0.046 [0.165]	
1–3 years	48	3.58 [7.16]		0.048 [0.074]	
>3 years	64	4.62 [6.33]	*0.96*	0.034 [0.060]	*0.61*
**Dominant site of relapse**					
Local regional	21	4.96 [10.53]		0.029 [0.021]	
Bone	101	4.90 [6.48]		0.044 [0.084]	
Other distant metastasis	66	3.43 [5.34]	***0.028***	0.050 [0.086]	*0.33*

Clinical parameters were analysed for their association with the gene expression of the two genes of interest; the median expression per gene including the interquartile range (IQR) and the number of patients per analysis (n) is shown. Pathological tumour size is defined as follows: pT1 < = 2 cm, pT2 > 2 cm and < = 5 cm, pT3 > 5 cm, and pT4 = tumour with direct extension to chest wall and/or skin. *Cut-offs for positive and negative hormone receptor/growth factor status were established as previously described^56,57^. *P* = p-value. Significant p-values are printed in bold.

#### *Hsa-miR-7, CDR1-AS* and the response to 1^st^-line tamoxifen therapy in the predictive cohort

We also investigated whether *hsa-miR-7* or *CDR1-AS* expression levels in the primary tumours had an influence on the type of response to tamoxifen in the predictive cohort (Table 5). To this end, we evaluated the contribution of our two markers using logistic regression analysis in a univariate model as well as a multivariate model. The latter model contained age at start of the 1^st^-line tamoxifen therapy, disease-free interval, dominant site of relapse, *ESR1* and *PGR* expression levels and *ERBB2* status.

**Table 5:** Uni- and multivariate association of response with *CDR1-AS*, *hsa-miR-7* and other clinical variables in the predictive cohort.

Parameters base model	univariate model				multivariate model		
	n	OR	(95% CI)	*P*	OR	(95% CI)	*P*
	188						
**Age at start 1** ^**st**^ **-line tamoxifen**							
≤55 years	75	1			1		
56–70 years	67	1.63	(0.82–3.25)	*0.16*	1.23	(0.55–2.72)	*0.61*
>70 years	46	3.06	(1.29–7.23)	***0.011***	2.05	(0.81–5.18)	*0.13*
**Disease-free interval**							
≤1 year	76	1			1		
1–3 years	48	1.52	(0.71–3.25)	*0.28*	1.44	(0.63–3.30)	*0.38*
>3 years	64	2.07	(0.99–4.28)	*0.05*	1.88	(0.86–4.14)	*0.12*
**Dominant site of relapse**							
Local regional	21	1			1		
Bone	101	0.25	(0.07–0.92)	***0.037***	0.20	(0.05–0.77)	***0.020***
Other distant metastasis	66	0.41	(0.11–1.56)	*0.19*	0.28	(0.07–1.19)	*0.08*
Hormone/Growth factor receptors*							
ER (log continuous *ESR1*)	188	1.65	(1.22–2.23)	***0.001***	1.65	(1.15–2.36)	***0.006***
PR (log continuous *PGR*)	188	1.07	(0.92–1.25)	*0.39*	1.00	(0.84–1.20)	*0.99*
HER2 (*ERBB2*) status							
Not amplified	165	1			1		
Amplified	23	0.60	(0.25–1.45)	*0.26*	0.97	(0.33–2.85)	*0.96*
					***hsa-miR-7*** **separately added to the base model**		
***CDR1-AS*** **(log continuous)**	188	1.01	(0.78–1.31)	*0.96*			
***hsa-miR-7*** **(log continuous)**	188	0.76	(0.60–0.96)	***0.023***	0.73	(0.57–0.95)	***0.017***
≤median	94	1			1		
>median	94	0.56	(0.30–1.03)	*0.064*	0.59	(0.30–1.16)	*0.13*

The association of the different parameters, including *CDR1-AS* and *hsa-miR-7* expression, with the therapy response in the predictive cohort is displayed on the left side of the table. The hormone/growth factor receptor expression values used in this table were determined by qPCR. * Cut-offs for positive and negative hormone receptor/growth factor status were established as previously described^56,57^. The same parameters are assessed with a multivariate model on the right side. n = number of patients, OR = odds ratio, CI = confidence interval, and *P* = p-value. Significant p-values are highlighted in bold.

We realised that the hazard ratios of the patients among the pre-menopausal patient age groups (≤55 years) were similar to one another, and the same was true for the hazard ratios for the post-menopausal patient age groups (>55 years). Due to this, the menopausal status was largely covered by the age groups, and due to the low patient numbers (n = 188) included in this cohort, we did not include menopausal status but rather the age categories in our analyses for the predictive cohort.

In the univariate analysis for response, only *hsa-miR-7* was a predictive biomarker, namely for a poor response to treatment, a finding which remained significant in the multivariate analysis (odds ratio 0.73, 95% CI 0.57–0.95, *P* = 0.017).

#### *Hsa-miR-7*, *CDR1-AS* and PFS in the predictive cohort

We subsequently investigated whether *hsa-miR-7* or *CDR1-AS* expression could predict the length of PFS (Table 6). In the Cox regression analysis, we evaluated the contribution of our two markers in a univariate model as well as in a multivariate model containing the same additional parameters as used for the response analysis.

**Table 6:** Uni- and multivariate association of *CDR1-AS*, *hsa-miR-7* and other clinical variables with PFS in the predictive cohort.

Parameters base model	univariate model				multivariate model		
	n	HR	(95% CI)	*P*	HR	(95% CI)	*P*
	188						
**Age at start 1** ^**st**^ **-line tamoxifen**							
≤55 years	75	1			1		
56–70 years	67	0.76	(0.54–1.07)	*0.11*	0.78	(0.54–1.13)	*0.19*
>70 years	46	0.57	(0.38–0.84)	***0.005***	0.65	(0.43–0.98)	***0.039***
**Disease-free interval**							
≤1 year	76	1			1		
1–3 years	48	0.79	(0.54–1.14)	*0.21*	0.83	(0.56–1.22)	*0.34*
>3 years	64	0.68	(0.48–0.97)	***0.032***	0.74	(0.51–1.05)	*0.09*
**Dominant site of relapse**							
Local regional	21	1			1		
Bone	101	1.60	(0.96–2.65)	*0.071*	1.99	(1.16–3.43)	***0.013***
Other distant metastasis	66	1.34	(0.78–2.27)	*0.28*	2.00	(1.10–3.63)	***0.023***
Hormone/growth factor receptors*							
ER (log continuous *ESR1*)	188	0.77	(0.67–0.89)	<***0.001***	0.82	(0.69–0.96)	***0.016***
PR (log continuous *PGR*)	188	0.89	(0.83–0.96)	***0.002***	0.92	(0.84–0.96)	***0.038***
HER2 (*ERBB2*) status							
Not amplified	165	1			1		
Amplified	23	1.86	(1.19–2.90)	***0.007***	1.49	(0.89–2.50)	*0.13*
					***hsa-miR-7*** **separately added to the base model**		
***CDR1-AS*** **(log continuous)**	188	0.97	(0.86–1.10)	*0.62*			
***hsa-miR-7*** **(log continuous)**	188	1.18	(1.04–1.33)	***0.008***	1.22	(1.08–1.38)	***0.002***
≤median	94	1			1		
>median	94	1.54	(1.14–2.09)	***0.005***	1.63	(1.18–2.25)	***0.003***

This table shows the associations of PFS with the cohort characteristics and *CDR1-AS* and *hsa-miR-7* expression. The different metrics were examined with an univariate analysis on the left side of the table, while they were analysed with a multivariate model on the right side. n = number of patients, HR = hazard ratio, CI = confidence interval, and *P* = p-value. Significant p-values are printed in bold. The hormone/growth factor receptor expression values used in this table were determined by qPCR. *Cut-offs for positive and negative hormone/growth factor status were established as previously described^56,57^.

**Figure 2:**
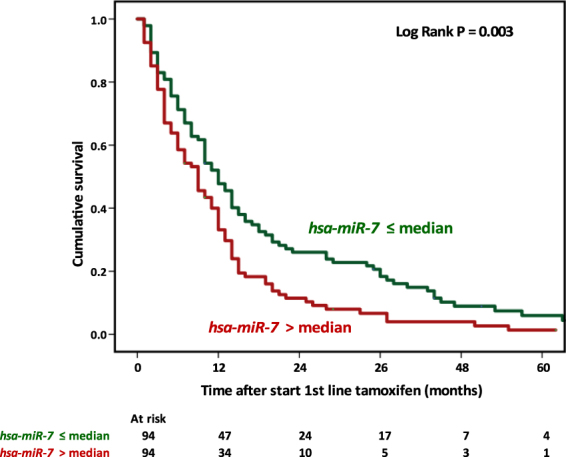
*Hsa-miR-7* as a function of progression-free survival. The figure shows *hsa-miR-7* expression split into two expression categories and its relationship with PFS in the predictive cohort in a Kaplan-Meier graph. The log rank test was applied to assess the significance and the obtained p-value is listed within the plot.

In the Cox univariate regression analysis, *hsa-miR-7*, but not *CDR1-AS*, was associated with the length of PFS and remained an independent predictive marker for poor PFS in the multivariate analysis (log continuous variable: HR = 1.22, 95% CI 1.08–1.38, *P* = 0.002; split on median: HR = 1.63, 95%CI 1.18–2.25, *P* = 0.003). The association of *hsa-miR-7* with PFS is visualised by a Kaplan-Meier plot after dividing our cohort into *hsa-miR-7*-high and -low subgroups based on the median expression level (Fig. 2).

#### *Hsa-miR-7* and *CDR1-AS* in regard to PR-OS in the predictive cohort

Finally, we related our markers to PR-OS after the beginning of tamoxifen therapy. While *CDR1-AS* did not show an association with PR-OS in the Cox univariate regression analysis, we did find an association between *hsa-miR-7* expression and PR-OS, which remained significant in the multivariate analysis (HR = 1.22, 95% CI 1.06–1.40, *P* = 0.004; split on median: HR = 1.64, 95% CI 1.17–2.29, *P* = 0.004 (Table 7).

**Table 7:** Uni- and multivariate association of post-relapse overall survival (PR-OS) with *CDR1-AS*, *hsa-miR-7* and other clinical variables in the predictive cohort.

Parameters	n	univariate model			multivariate model		
		HR	(95% CI)	*P*	HR	(95% CI)	*P*
	188						
**Age at start 1** ^**st**^ **-line tamoxifen**							
≤55 years	75	1			1		
56–70 years	67	1.04	(0.72–1.48)	*0.85*	0.98	(0.65–1.47)	*0.93*
>70 years	46	0.94	(0.62–1.45)	*0.79*	1.10	(0.70–1.73)	*0.69*
**Dominant site of relapse**							
Local regional relapse	21	1			1		
Bone metastasis	101	1.63	(0.94–2.83)	*0.08*	1.61	(0.92–2.84)	*0.10*
Other distant metastasis	66	1.55	(0.87–2.75)	*0.14*	1.81	(0.99–3.31)	*0.06*
**Disease-free interval**							
≤1 year	76	1			1		
1–3 years	48	0.83	(0.56–1.22)	*0.34*	0.93	(0.62–1.41)	*0.75*
>3 years	64	0.56	(0.38–0.82)	***0.003***	0.56	(0.38–0.85)	***0.006***
Hormone/Growth factor receptors*							
ER (log continuous *ESR1*)	188	0.80	(0.68–0.93)	***0.005***	0.79	(0.66–0.95)	***0.012***
PR (log continuous *PGR*)	188	0.88	(0.81–0.95)	***0.001***	0.90	(0.82–0.98)	***0.020***
HER2 (*ERBB2*) status							
Not amplified	165	1			1		
Amplified	23	1.45	(0.91–2.30)	*0.12*	1.15	(0.69–1.93)	*0.59*
					***hsa-miR-7*** **separately added to the base model**		
***CDR1-AS*** **(log continuous)**	188	0.97	(0.83–1.13)	*0.72*			
***hsa-miR-7*** **(log continuous)**	188	1.22	(1.07–1.39)	***0.004***	1.22	(1.06–1.40)	***0.004***
≤median	94	1			1		
>median	94	1.70	(1.23–2.35)	***0.001***	1.64	(1.17–2.29)	***0.004***

The association of *CDR1-AS* and *hsa-miR-7* expression and other clinical variables with PR-OS using the univariate model is shown on the left side and with the multivariate model on the right side. The hormone receptor/growth factor expression values used in this table were determined by qPCR. *Cut-offs for positive and negative hormone receptor/growth factor status were established as previously described^56,57^. n = number of patients, HR = hazard ratio, CI = confidence interval, and *P* = p-value. Significant p-values are highlighted in bold.

## Discussion

Our study showed among the first results an inverse relationship between *CDR1-AS* and *hsa-miR-7* expression. This finding is supported by previous studies in different cancer patient cohorts^25,26^. Besides, we observed an inverse relationship between *hsa-miR-7* and *EGFR*. This observation is in line with the known function of *hsa-miR-7* to target the *EGFR* mRNA 3′ UTR and cause the downregulation of *EGFR* mRNA in this way^22^. Due to the inverse relationship of *hsa-miR-7* with *CDR1-AS, CDR1-AS* can be expected to be expressed at higher levels in *EGFR*-high tumours, and this was the case in our dataset.

One point of caution is, however, that *EGFR* is not very highly expressed in most breast tumour cells^27–29^. As tumours consist not only of tumour cells, but also of stroma and immune cells^30^, *CDR1-AS* might be mainly expressed by non-tumour cell types. Supporting this hypothesis, we found that *CDR1-AS* was associated with tumours with a lower tumour cell content, and in our cell line expression study, *CDR1-AS* expression was amongst the highest in the stromal fibroblast strain (Fig. 1). However, higher expression in immune cells infiltrating the tumours (tumour-infiltrating leukocytes; TILs) could also be a possible explanation. Therefore, we assessed several immune cells (CD4+ T helper cells, CD8+ T helper cells, CD56+ granulocytic NK cells and HLA-DR+ antigen-presenting cells (APC)) for *CDR1-AS* expression in a healthy donor and found low *CDR1-AS* expression. To further examine the relationship of *CDR1-AS* expression with stroma, we performed correlations for *hsa-miR-7* and *CDR1-AS* with several stromal markers^30–33^, a newly developed stromal gene expression signature and our developed TIL signature, which is a previously published gene expression signature for tumour-infiltrating leukocytes^34,35^ (see also Supplementary Methods). The TIL signature was established on breast tumours and was developed based on differential gene expression between breast tumours with pathologically high and low TIL scores and validated on an large independent cohort (data not shown)^34,35^. The correlation analyses showed that tumour cell content was strongly negatively correlated with the TIL signature (Spearman rs = −0.428; P = 2.47E-12), but showed slightly lower negative correlations with the set of individual stromal markers and the stromal signature (Supplementary Table S3). This indicates that the majority of non-tumour cells in our tumour samples were mainly TILs and not stromal cells. That said, in our cohort, *CDR1-AS* correlated very strongly positively with the stromal signature (Spearman rs = 0.674, *P* = 8.30E-34) and the stromal markers (Spearman rs = 0.25 up to 0.53; all *P* < 1E-05; see Supplementary Table S3 for details), but not with the TIL signature (*P = *0.16). Based on these results we conclude that *CDR1-AS* appears to be mainly expressed by stromal cells and not by TILs or tumour cells.

*Hsa-miR-7*, was also not associated with the TIL signature. Furthermore, *hsa-miR-7* was correlated negatively with the stromal signature as well as with the analysed stromal markers. In contrast *hsa-miR-7* showed a positive association with the tumour cell content in the primary tumours from our recurrent breast cancer cohort (predictive cohort). These associations support the expression of *hsa-miR-7* mainly by tumour cells.

Based on our correlative findings, it could be speculated that *CDR1-AS*’ role is to prevent *hsa-miR-7* from being active in the stroma but future studies exploring this result in more detail are needed.

In our prognostic cohort, we observed that *hsa-miR-7* expression was associated with tumour size, tumour grade, ER status and PR status. Looking at the expression levels of this microRNA, it displayed higher expression in the prognostic-wise worse parameter categories, such as pT3 and pT4 size status, poor tumour grade, ER-negative tumours and PR-negative tumours. Based on these findings, *hsa-miR-7* appears to be associated with a generally more aggressive tumour type. Indeed, with respect to the prognosis of breast cancer patients with LNN disease who did not receive systemic adjuvant therapy, we confirmed a shorter MFS for patients with higher *hsa-miR-7* levels in ER-positive tumours, which is in line with our previous findings in a subset from this cohort^21^. Surprisingly, although overall *hsa-miR-7* expression was higher in ER-negative tumours, this microRNA was not associated with prognosis in this subgroup. However, the microRNA was related to prognosis in ER-positive tumours, indicating that these tumours, despite being ER-positive, have more aggressive features similar to ER-negative tumours.

While *CDR1-AS* did not appear to correlate with either ER or PR expression, we did observe several associations for *CDR1-AS* with clinical parameters in our LNN cohort (e.g., tumour size and grade) which were inverse to those found for *hsa-miR-7*. Nevertheless, our data showed that the expression of this circRNA is not significantly (*P* > 0.05) related to relevant parameters such as hormone receptor expression or clinical outcomes; therefore, it is likely that factors other than sequestering *hsa-miR-7* play a more prominent role here.

In our cohort of patients who received 1^st^-line tamoxifen treatment, we observed that high *hsa-miR-7* levels were associated with a shorter time to progression, which remained significant after adjustment for known predictive factors in breast cancer. This finding indicates that high *hsa-miR-7* levels are an independent predictor for shorter PFS. Furthermore, *hsa-miR-7* was also associated with a worse clinical response to 1^st^-line tamoxifen therapy. These observations not only confirm the general notion that *hsa-miR-7* is a marker of more aggressive tumours but also that *hsa-miR-7* is a predictor of poor tamoxifen therapy efficacy, irrespective of the intrinsically aggressive tumour type.

In addition to the associations of *hsa-miR-7* with therapy response and progression, we also found an association with survival. The association with OS was weak in our prognostic cohort and only present in the ER-positive subset (*P* = 0.041), while in the predictive cohort, *hsa-miR-7* was stronger associated with PR-OS (*P* = 0.004). These findings exemplify the potential of this microRNA as a biomarker in breast cancer.

Regarding the regulation of *CDR1-AS* and *hsa-miR-7* expression, it has been found that the upregulation of c-Myc causes *CDR1-AS* downregulation in B cells, although it remains unclear whether c-Myc can bind the *CDR1-AS* promoter region directly^36^. Interestingly, it has been shown that c-Myc upregulates *hsa-miR-7* expression^18,37^. This might indicate an additional level of regulation for both genes by c-Myc and the subsequent fine tuning of the RNA levels for these two genes through their interplay. One could hypothesise that *hsa-miR-671*, a negative regulator of *CDR1-AS*^38^, could be upregulated by c-Myc and in this way also downregulate *CDR1-AS* levels; however this does not seem to be the case as *hsa-miR-671* levels have been investigated upon c-Myc induction and no change in expression was found^39^.

Recently, several studies showed an association of *CDR1-AS* with clinical outcomes in different cancer types. Two studies reported that the circRNA was associated with poor survival in colorectal cancer patients^25,40^, a cancer-type in which low *hsa-miR-7* expression has previously been associated with poor survival^41^. Interestingly there is a molecular subtype in colorectal cancer (CMS4) that is characterised by stromal invasion and has been linked to a worse overall survival, relapse-free survival and is frequently diagnosed at a higher stage^42^. This might explain the differential outcomes between our study and the previous colorectal cancer studies linking *CDR1-AS* to worse outcomes^25,40^, if indeed subtype CMS4 is well-represented in those cohorts, which would also support a link between *CDR1-AS* expression and stroma and, in colorectal cancer, poor outcome.

Another study on gastric cancer also found an association of increased *CDR1-AS* expression with poor survival^43^. In hepatocellular carcinoma, the situation is less clear; while *CDR1-AS* was associated with several markers of poor prognosis, it was not significantly associated (*P* > 0.05) with recurrence^26^.

Overall, there have been conflicting reports on *hsa-miR-7*, labelling it as an oncomir on the one hand and as a tumour suppressor microRNA on the other hand. Several cell line and animal studies support its tumour suppressor role with a specific focus on the target *EGFR*^44,45^; associated proteins, such as *PAK1*^46,47^; as well as transcription factors, such as *YY1*^20^. By contrast, others have found oncogenic roles for *hsa-miR-7* by targeting a transcriptional repressor^18^ or the tumour suppressor *KLF4*^48^. The associations with clinical outcomes in patients are conflicting for this microRNA as well. On the one hand, it has been found that lower *hsa-miR-7* expression is associated with poor survival in gastric cancer (n = 106)^49^ and with more metastases and a shorter OS in lung cancer patients (n = 108)^50^, indicating a tumour suppressor role of the microRNA. On the other hand, arguing for an oncogenic role, a study on colorectal cancer patients (n = 210) found an association of high *hsa-miR-7* levels with several clinical markers for poor prognosis, different relapse types and lower overall survival^51^. Furthermore, a study on prostate cancer patients (n = 45) also showed that increased expression of this microRNA is associated with a worse OS. In the latter study, *hsa-miR-7* expression was also linked to a shorter time span until the tumours became castration-resistant^52^.

In the case of breast cancer, there has been one report associating lower *hsa-miR-7* expression levels with lymph node-positive breast cancer patients versus lymph node-negative patients^53^. However, in this small study, neither the tumour ER status nor information on potential treatments before surgery or adjuvant treatment were reported^53^. Those factors could introduce a bias, especially when the patient numbers are small, and this could explain the different results from our study.

In general, the contradictions in different studies regarding the role of *hsa-miR-7* might indicate that this microRNA is co-regulated with different causal factors making it a biomarker of aggressive tumours in one cancer and a biomarker of less aggressive tumours in another. Further studies with large well-characterised patient cohorts as well as functional studies are needed to clarify the contradictions with regard to this microRNA and patient outcome.

Based on our findings in a 1^st^-line tamoxifen-treated patient cohort, we conclude that *hsa-miR-7* is a predictive biomarker for poor response and PFS. Our previous study showed that *hsa-miR-7* was a prognostic biomarker in a LNN breast cancer cohort^21^, and the present study confirmed this result in an, albeit not fully independent, extended LNN breast cancer cohort. Additionally, *hsa-miR-7* was found to be associated with short OS in the ER-positive subset in the prognostic cohort and PR-OS in the predictive cohort. By contrast, *CDR1-AS* did not show associations with any clinical outcomes in our breast cancer cohorts.

In conclusion, we showed that in addition to the known association between high *hsa-miR-7* expression and a worse course of disease (earlier development of metastases) in primary breast cancer, high *hsa-miR-7* levels are predictive of an adverse response to tamoxifen therapy and poor progression-free and post-relapse overall survival in patients with recurrent disease. These associations do not appear to be related to *CDR1-AS*. However, in light of recent reports describing the clinical utility of *CDR1-AS* expression in other cancer types^25,26,40,43^, it will be interesting to study this gene further, including its stromal location, in large cohorts focused on those cancer types and to investigate its functional influence on cancer progression.

## Material and Methods

### Patient samples

The protocol to study biological markers associated with disease outcome was approved in writing by the medical ethics committee of the Erasmus Medical Centre Rotterdam, The Netherlands (MEC 02.953) and was performed in accordance with the Code of Conduct of the Federation of Medical Scientific Societies in The Netherlands (https://www.federa.org/codes-conduct). The use of anonymous or coded left over material for scientific purposes is part of the standard treatment agreement with patients and therefore informed consent was not required according to Dutch law. The results of this study are reported based on the REMARK criteria for clinical reporting^54^.

For our analyses we generated *CDR1-AS* and *hsa-miR-7* expression data using qPCR (see below) for two different clinical cohorts. These assays were performed blinded to the study endpoint.

In this retrospective study, tumours of female patients were included, who underwent surgery for invasive primary breast cancer between 1980 and 1995 in the Netherlands. A further selection criterion was no previously diagnosed cancers with the exception of basal cell carcinoma or stage Ia/Ib cervical cancer.

Within this study only data from sections of primary tumours with at least 30% invasive tumour cells, evaluated as described before^55^, were included. The primary tumour specimens used in this study for RNA isolation consisted of fresh frozen tissue that had been stored in liquid nitrogen. At least 100 mg of tumour material per patient had to be available. The details of tissue processing have been described previously^55^.

No stratification or matching was used for our cohorts. The tumour grade was assessed according to standard procedures at the time of inclusion.

For the classification of patients’ RNA samples regarding ER expression, PR expression and HER2 amplification status, RT-qPCR was used with the following cut-offs: (1) ER (*ESR1* mRNA) 0.2, (2) PR (*PGR* mRNA) 0.1 and (3) HER2 (*ERBB2* mRNA) 18; the cut-offs were established as previously described^56,57^.

The first cohort (prognostic cohort) included primary tumours from LNN breast cancer patients who had not received any systemic (neo)adjuvant therapy. The different inclusion criteria discussed within this section led to a cohort consisting of 120 ER-negative and 225 ER-positive primary tumours. The median age of the patients in the study at time of surgery was 55 years (ranging from 27–88 years). The median follow-up time of living patients was 91 months (ranging from 8–319 months). MFS was defined as the time between surgery and the development of a distant metastasis. One-hundred-seventy (49%) patients developed a distant metastasis that counted as an event in the MFS analysis. Patients who died without evidence of disease were censored at the last follow-up in the MFS and OS analyses. In total, 178 patients were censored in the OS analysis and 175 were censored in the MFS analysis. Additional patient and tumour characteristics are presented in Table 1.

The second cohort (predictive cohort) consisted of 188 hormone-naïve breast cancer patients with ER-positive primary tumours who received tamoxifen as a 1^st^-line treatment for recurrent disease and fulfilled our inclusion criteria (discussed above). Thirty-five of the patients received adjuvant chemotherapy. The 188 patients [21 patients with local-regional relapse (LRR) and 167 patients with a distant metastasis] were treated with tamoxifen (40 mg daily) therapy. The median age of the patients at the start of tamoxifen therapy was 61 years (ranging from 29–90 years). We used the primary tumours of these patients for our analyses. The median time between primary surgery and the start of therapy was 27 months (ranging from 2–115 months). To evaluate PFS, the start of the 1^st^-line tamoxifen therapy was set as zero and the end point was at the time of progression or the last date of follow-up. The median follow-up time for living patients at the end of follow-up was 64 months (ranging from 8–272 months) after primary surgery and 10 months (ranging from 1–144 months) after the start of tamoxifen therapy. The disease-free interval was defined as the interval between the breast cancer diagnosis and the first recurrence of the disease. At the end of the follow-up period, 178 (95%) patients had developed tumour progression and 154 (82%) patients had died. In the PFS analysis, 10 patients were censored, while 34 patients were censored in the PR-OS analysis. Clinical response to tamoxifen therapy was defined by standard Unio Internationale Contra Cancrum criteria^58^ and described previously^55^. Those 126 patients with an evident tumour reduction of 50% or more (partial and complete remission) or no change in tumour volume after more than 6 months were defined as responders to 1^st^-line tamoxifen therapy.

Non-responders included the 62 patients who showed tumour progression (progressive disease) or no change in tumour volume after ≤6 months. The median PFS times were as follows: (1) complete remission (n = 7), 16 months; (2) partial remission (n = 34), 15 months; (3) stable disease (n = 85), 14 months; (4) no change after ≤6 months (n = 9), 5 months; and (5) progressive disease (n = 53), 3 months. Additional patient and tumour characteristics for this cohort are shown in Table 3.

An overview of the specifics for the clinical cohorts and cell lines [see below] are depicted in a flow diagram in Supplementary Fig. S1.

### Cell lines, immune cells and tumour pool used in the *CDR1-AS* expression experiment

For our cell line experiments, we used total RNA isolated from breast cancer cell lines with different intrinsic subtype characteristics described previously^59,60^ (see Supplementary Table S4 for cell lines, subtypes and normalised *CDR1-AS* expression values) supplemented with total RNA isolated from the endothelial cell line EA.hy926 and a fibroblast strain (M92–19T) derived from a primary breast cancer tumour^21^. Furthermore we included MACS sorted immune cell subsets from PBMCs which were isolated from buffy coats by Ficoll gradient centrifugation from a healthy donor. All cultured cell lines were established to be of correct identity by performing STR profiling analyses using the PowerPlex® 16 system (Promega, Madison, WI, USA). The breast tumour pool used in this experiment consisted of cDNA from 100 primary breast tumours from lymph node-negative and -positive patients who did not receive neo-adjuvant treatment. Overall, the samples were *ESR1*-positive, *PGR*-positive, had no *ERBB2* amplification and had a high proliferative index [GGI; 1.1]^61^.

### MACS

Magnetic-activated cell sorting (MACS) was performed using the MACS technology with an anti-PE positive selection kit (Miltenyi Biotec, Leiden, the Netherlands) according to the manufacturer’s protocol. Briefly, 5E06 PBMC in 100 μL MACS buffer were stained with 10 μL PE-labelled primary antibody conjugate (CD4, CD8, CD56 or HLA-DR) and incubated for 10 minutes at 6–8 °C, in the dark. Following washing with MACS buffer the cells were labelled with 20 μL anti-PE magnetic beads for 15 minutes at 6–8 °C in the dark. After additional washing, the cells were applied to MACS MS columns which were placed in a magnetic field. Finally, the positively selected cell fraction was eluted, cells were counted and the purity (percentage of PE labelled cells) was analysed by flow cytometry using a BD FACSCelesta (BD Biosciences, San Jose, CA, USA). Purities of the cell sorting per targeted immune cell population are given in Supplementary Table S5.

### RNA isolation, cDNA synthesis and quantitative real time PCR (RT-qPCR)

Tissue processing, total RNA isolation and total RNA quality control checks have been described elsewhere^55,62^. Briefly, total RNA was isolated with RNA Bee (Thermo Fisher Scientific, Waltham, MA, USA) from 30 µm tissue sections. MRNAs/circRNAs were reverse-transcribed with the RevertAid H Minus First Strand cDNA Synthesis Kit from Thermo Fisher Scientific, followed by an RNAse H step (Ambion, Thermo Fisher Scientific). QPCR reactions were performed in a 25 µL final volume using an Mx3000P^TM^ Real-Time PCR System (Agilent Technologies, Amstelveen, the Netherlands). All qPCR assays were established to have an efficiency of at least 90% and did not generate a product within 35 cycles in the absence of reverse transcriptase. Three reference genes were selected based on the literature and tested for their signal intensity and stable expression level between both LNN and lymph-node positive (LNP) as well as ESR1-high and ESR1-low samples using GeNorm^63^ and NormFinder^64^ software packages available in GenEx 6.1.1.550 (MultiD Analyses AB, Gotenborg, Sweden). mRNA/circRNA levels were assessed relative to the average expression of *HMBS*, *HPRT1* and *TBP* using the delta Cq method (dCq = 2^(average Cq reference genes - Cq target gene)). *Hsa-miR-7* levels were measured and quantified relative to *hsa-miR-132* and *hsa-miR-374* as described previously^21^. For the cultured cell lines and clinical specimen we incorporated only samples in our study in which we could detect the reference gene *HMBS* within 25 qPCR cycles when using 10 ng of total RNA as input. This to ensure only RNA samples with sufficient quantity and of good quality are measured.

All of the primers and hydrolysis probe assays used for gene expression are shown in Supplementary Table S6.

### Figure preparation and statistical analysis

Figures were generated using SPSS version 24 (IBM, Armonk, NY, USA), Excel (Microsoft Corporation, Redmond, WA, USA) and/or Inkscape (Free Software Foundation Inc., Boston, MA, USA). All statistical analyses were performed in SPSS (version 24). Cox univariate and/or multivariate regression analysis were used for the survival analyses (MFS, PFS, OS and PR-OS). MFS was defined as the time between the primary surgery and the first distant metastasis. PFS was defined as the time between the start of the first line therapy (tamoxifen) and the first sign of progression. OS was defined as the time between the primary surgery and death or the last follow up. PR-OS was defined as the time between relapse and death or the last follow-up. Mann-Whitney tests were used to analyse *CDR1-AS* and *hsa-miR-7* with clinical parameters (except for cases when more than 2 groups were analysed; then a Kruskal-Wallis test was performed); the clinical parameters were used as grouping variables. Logistic regression was used to assess whether treatment responses were associated with *CDR1-AS* and *hsa-miR-7* expression. The non-parametric Kaplan-Meier estimator was used to estimate and plot the survival functions, with the log-rank test to assess for differences. All statistical tests were two-sided and *P* < 0.05 was considered statistically significant.

### Data availability

The full data sets used in this paper are available upon request.

## Electronic supplementary material

Supplementary info

Supplementary Tables 1 to 6
