# Supplementary material for: Association of microRNA-7 and its binding partner CDR1-AS with the prognosis and prediction of 1st-line tamoxifen therapy in breast cancer
Source: Sci Rep. 2018 Jun 25;8:9657. doi: 10.1038/s41598-018-27987-w (PMC6018428; doi:10.1038/s41598-018-27987-w)
Supplement: Supplementary file 1 — Supplementary info [file 41598_2018_27987_MOESM1_ESM.pdf]

Association of microRNA-7 and its binding partner *CDR1-AS* with the prognosis and prediction of 1st-line tamoxifen therapy in breast cancer

K. Uhr, A.M. Sieuwerts, V. de Weerd, M. Smid, D. Hammerl, J.A. Foekens, J.W.M. Martens

Supplementary methods

Stromal signature

To obtain a stroma-specific gene expression signature public data were downloaded from the Gene Expression Omnibus using accession GSE5847. This data was generated within a study of Boersma *et al.* (Boersma BJ *et al*.: A stromal gene signature associated with inflammatory breast cancer. Int J Cancer 2008 Mar 15;122(6):1324-32) and had been used in the following study of Martin *et al.* (Martin DN *et al.*: Differences in the tumor microenvironment between African-American and European-American breast cancer patients. PLoS One 2009;4(2):e4531). On this dataset a paired t-test was performed to detect those genes which were significantly higher expressed in microdissected stroma (FDR<0.05 and fold-change > 1.7).

The average expression of all signature-genes per sample was then correlated to the expression of the respective parameter to obtain the associations listed in **Supplementary Table 3.**

Supplementary figures

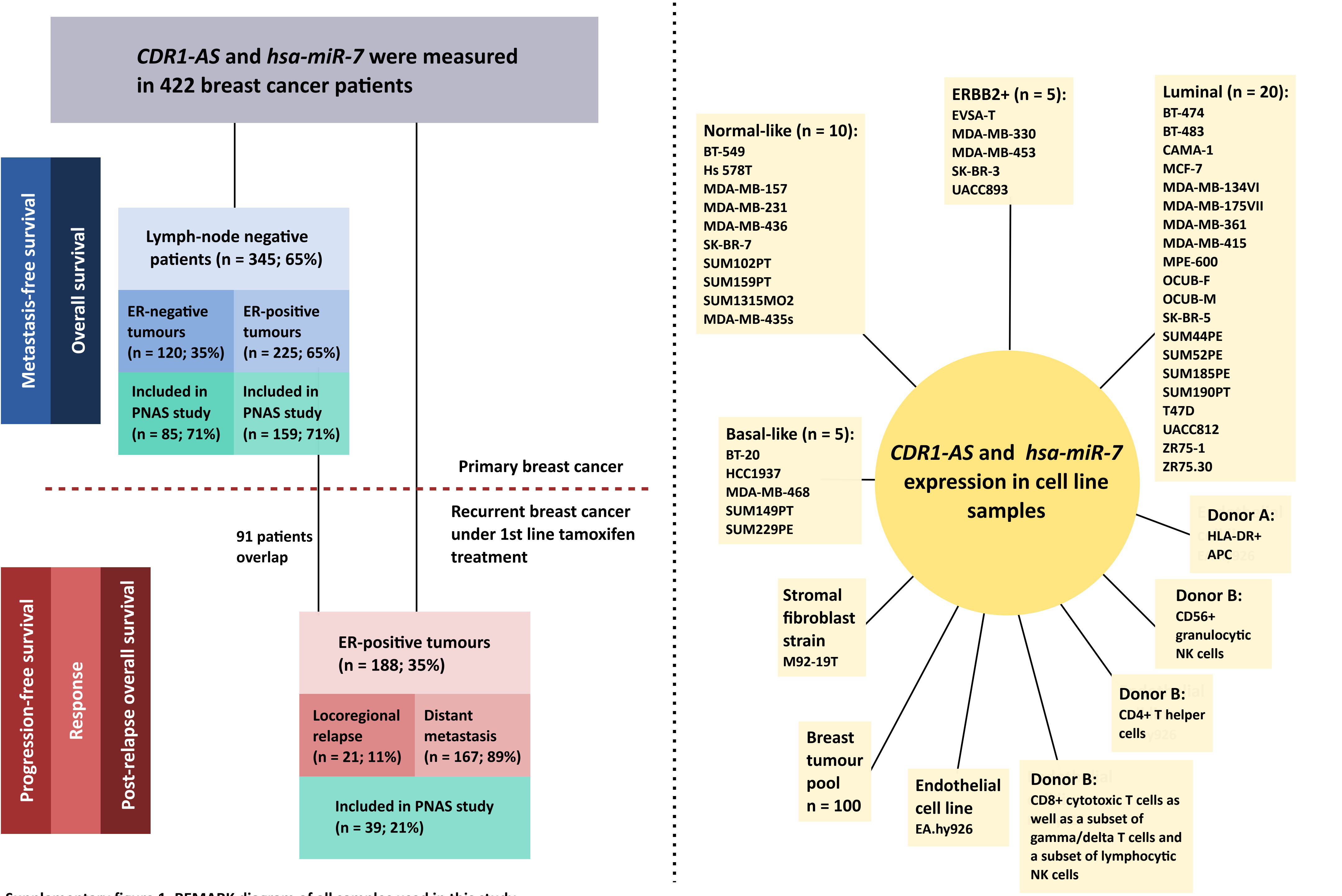

Supplementary figure 1. REMARK diagram of all samples used in this study

On the left side of the figure the patient numbers are shown for each cohort as well as the number of patients that overlapped between the two cohorts and with an earlier study (PNAS study). Furthermore the main outcomes studied per cohort are shown as well as some cohort characteristics (ER status, type of relapse). On the right side the cell line samples used in this study are listed per subtype.
